# Energy Dynamics in the Brain: Contributions of Astrocytes to Metabolism and pH Homeostasis

**DOI:** 10.3389/fnins.2019.01301

**Published:** 2019-12-06

**Authors:** Joachim W. Deitmer, Shefeeq M. Theparambil, Ivan Ruminot, Sina I. Noor, Holger M. Becker

**Affiliations:** ^1^Department of Biology, University of Kaiserslautern, Kaiserslautern, Germany; ^2^Centre for Cardiovascular and Metabolic Neuroscience, Department of Neuroscience, Physiology and Pharmacology, University College London, London, United Kingdom; ^3^Centro de Estudios Científicos, Valdivia, Chile; ^4^Centre for Organismal Studies, Heidelberg University, Heidelberg, Germany; ^5^Institute of Physiological Chemistry, University of Veterinary Medicine Hanover, Hanover, Germany

**Keywords:** lactate, monocarboxylate transporters, carbonic anhydrases, protons, bicarbonate, glycolysis

## Abstract

Regulation of metabolism is complex and involves enzymes and membrane transporters, which form networks to support energy dynamics. Lactate, as a metabolic intermediate from glucose or glycogen breakdown, appears to play a major role as additional energetic substrate, which is shuttled between glycolytic and oxidative cells, both under hypoxic and normoxic conditions. Transport of lactate across the cell membrane is mediated by monocarboxylate transporters (MCTs) in cotransport with H^+^, which is a substrate, a signal and a modulator of metabolic processes. MCTs form a “transport metabolon” with carbonic anhydrases (CAs), which not only provide a rapid equilibrium between CO_2_, HCO_3_^–^ and H^+^, but, in addition, enhances lactate transport, as found in *Xenopus* oocytes, employed as heterologous expression system, as well as in astrocytes and cancer cells. Functional interactions between different CA isoforms and MCTs have been found to be isoform-specific, independent of the enzyme’s catalytic activity, and they require physical interaction between the proteins. CAs mediate between different states of metabolic acidosis, induced by glycolysis and oxidative phosphorylation, and play a relay function in coupling pH regulation and metabolism. In the brain, metabolic processes in astrocytes appear to be linked to bicarbonate transport and to neuronal activity. Here, we focus on physiological processes of energy dynamics in astrocytes as well as on the transfer of energetic substrates to neurons.

## Energetic Requirements of Brain Cells

The human brain is a high-energy consuming organ. The requirement for energy supply is particularly high in many neurons for appropriate functioning of these cells (Collins, 1991; Attwell and Laughlin, 2001). A large portion of this energy is consumed at synapses, since electrical potential changes at postsynaptic sites are based on sustained ion gradients, which are based mainly on a large Na^+^ gradient, which is maintained by the activity of the Na^+^/K^+^ ATPase (the Na-K pump). The maintenance of a steep Na^+^ gradient across the cell membrane ensures also the regulation of other ions, such as Ca^2+^, Mg^2+^, H^+^, HCO_3_^–^ and Cl^–^, which are transported with, or exchanged by, Na^+^ in order to maintain their physiological levels in the cells (and sometimes also outside cells). The high energy requirement results from the large number of neurons (in mammals in the order of 10^10^ to 10^11^, which each can have hundreds or thousands of synapses; in some cases, like in the cerebellum, Purkinje cells contain synapses in the order of 10^5^), which can be active with tens or hundreds of signals per second at a given time. Even though each potential may be based on a very small current flow across the membrane (in the order of 10^–9^ A), it is the large quantity of potentials, which can be evoked in the enormous number of synapses (altogether estimated up to 10^15^ in the human brain), even if only a small fraction of those may be active at high frequency at a given time.

Another challenge for the supply of sufficient energy in the brain is that by far not every cell can be reached directly by capillaries (Mathiisen et al., 2010). In fact, there can be 3–6 cell layers between two blood capillaries. This requires other pathways for energetic compounds to individual cells, which may be coined “parenchymal diffusion.” However, this process can be slow, and in high-energy hotspots, too slow to supply enough energy to the cells. Microanatomical analysis revealed that blood capillaries, which deliver glucose, are covered to a large extent by astrocytic endfeet (Mathiisen et al., 2010), which allow the rapid uptake of this energetic compound into astrocytes. In fact, while the concentration of glucose in the blood capillaries is 3–6 mM, the concentration of glucose in the brain parenchyma can be as low as 0.5–1 mM (Dienel, 2019), indicating that most of the glucose is presumably taken up by astrocytes, before it is transported into the brain parenchyma. Since glucose, which is efficiently taken up by facilitated diffusion, is quickly phosphorylated in cells, there is always a steep gradient of glucose into the cells, even at extracellular concentration of 1 mM and below. This implies that neurons, which can be tens of microns away from the next blood vessel, can still take up glucose in considerable amounts. Nevertheless, the anatomical peculiarity giving astrocytes, which are believed to require much less energy to function physiologically than neurons, direct access to the vasculature, may hint to special cellular strategies to support energy supply to neurons under conditions of a high-energy demand (Magistretti et al., 1999). This strategy could imply that astrocytes transfer part of the energetic material they have exclusively direct access to, to neurons and hence play a role as mediators for additional energy supply to neurons (Bélanger et al., 2011).

## Metabolism in Brain Cells

**FIGURE 1:**
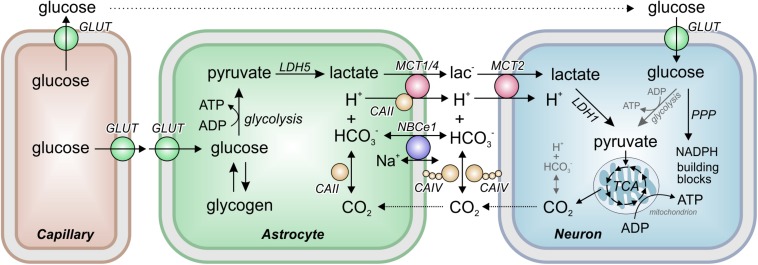
The Astrocyte to Neuron Lactate Shuttle. Astrocytes take up glucose from the blood capillaries via glucose transporters (GLUTs). In astrocytes, glucose is either stored as glycogen or metabolized to pyruvate in the glycolysis. Pyruvate is then converted to lactate by the oxidoreductase lactate dehydrogenase (LDH) isoform 5 (LDH5). The lactate is transferred from astrocytes to neurons by the monocarboxylate transporters (MCTs) MCT1, MCT2, and MCT4 in cotransport with a proton. MCT transport activity was found to be facilitated by interaction with the carbonic anhydrases (CAs) CAII and CAIV, which catalyze the equilibrium of H^+^, HCO_3_^–^ and CO_2_ both intra- and extracellularly, and by the activity of the electrogenic sodium-bicarbonate cotransporter NBCe1. In neurons, lactate is converted back to pyruvate by LDH1 and transferred into mitochondria for aerobic energy production in the tricarboxylic acid cycle (TCA). In addition, glucose is directly taken up into neurons where it can either serve as energy source in the glycolysis or is shuttled into the pentose phosphate pathway (PPP) for production of NADPH and cellular building blocks like ribose-6-phosphate.

The major energy source for the brain is glucose. Indeed, the human brain consumes approximately 20% of the glucose-derived energy (5.6 mg/100 g of brain tissue; Erbsloh et al., 1958). Glucose, which is taken up by facilitated diffusion via glucose transporters (GLUTs), can either be stored as glycogen (in the brain, the major glycogen stores are found in the astrocytes) or metabolized in the glycolytic pathway (Figure 1). The final product of glycolysis is pyruvate, which is either transferred into mitochondria, where it is metabolized in the citric acid cycle, or converted to lactate. Conversion of pyruvate to lactate is catalyzed by the oxidoreductase lactate dehydrogenase (LDH), which reduces pyruvate to lactate and oxidizes NADH + H^+^ to NAD^+^ (thereby lactate production results in the consumption of protons). The reaction is reversible, allowing cells to either produce or consume lactate, depending on their metabolic profile.

According to the “Astrocyte-to-Neuron Lactate Shuttle” (ANLS) hypothesis (Magistretti et al., 1999), lactate is primarily produced by astrocytes. From there, lactate is transferred via monocarboxylate transporters (MCTs) to neurons, where it is converted to pyruvate for aerobic energy production in mitochondria (Figure 1). Hence, additional pyruvate is generated, and precious reduction equivalents are produced in form of NADH during the conversion of lactate into pyruvate in neurons. A more detailed account on brain energy metabolism with respect to functional imaging studies has been contributed by Magistretti and Allaman (2015). The ANLS hypothesis is, though backed by multiple evidence, still controversial (Yellen, 2018; Dienel, 2019). Neurons themselves can have an increased glycolysis when stimulated and even release lactate (Dienel, 2017; Díaz-García and Yellen, 2019). It should be kept in mind that different metabolic conditions may indeed challenge rather different strategies concerning glucose and lactate flow in and between neurons and astrocytes, and that ANLS may only be one of several ways to meet the energy requirements of cells under different demands.

## Lactate Transport Between Brain Cells and Its Regulation

Lactate is transferred between astrocytes and neurons by MCTs. The MCTs belong to the SLC16 gene family, which comprises 14 isoforms, the first four of which (MCT1-4) transport lactate, but also other monocarboxylates, like pyruvate and ketone bodies, in cotransport with H^+^ in an electroneutral 1:1 stoichiometry (Halestrap and Price, 1999). In the brain, MCT1 is mainly expressed in endothelial cells and astrocytes (Bergersen et al., 2001; Pierre and Pellerin, 2005). MCT1 has a K_m_ for lactate of 3–5 mM (Bröer et al., 1997, 1998) and was therefore suggested to serve both as a lactate importer and exporter, depending on the cell’s metabolic profile. MCT2, which is a high-affinity transporter, with a *K*_m_ value of 0.7 mM for lactate (Bröer et al., 1999), is expressed primarily in neurons, where it mediates the uptake of lactate (Bergersen et al., 2001; Chiry et al., 2008; Mazuel et al., 2017). MCT3, which is exclusively expressed in the basolateral membrane of retinal pigment epithelium cells, has a *K*_m_ value for lactate of 6 mM (Grollman et al., 2000) and was suggested to mediate H^+^/lactate export from the retina (Deora et al., 2005; Daniele et al., 2008). MCT4 is a low-affinity, high-capacity transporter, with a *K*_m_ value for lactate of 20–37 mM (Dimmer et al., 2000). MCT4 is strongly expressed in astrocytes, where it serves as lactate exporter (Bergersen et al., 2001; Pierre and Pellerin, 2005). MCT-mediated lactate transport has been attributed a fundamental role in many (patho-)physiological processes in the brain. Lactate contributes to ion homeostasis and synaptic activity (Angamo et al., 2016) and can be essential for long-term memory formation (Suzuki et al., 2011), and promotes adult hippocampal neurogenesis (Lev-Vachnish et al., 2019). Furthermore, lactate mediates neuroprotective effects following traumatic brain injury (Álvarez et al., 2014; Zhou et al., 2018). Besides lactate, other MCT substrates could play a role in the modulation of neuronal activity and homeostasis of the CNS. MCT-mediated transport of β-hydroxybutyrate, for instance, which was attributed anti-epileptic effects (Lutas and Yellen, 2013) was shown to modulate neuronal excitability both under normal and ketogenic conditions (Valdebenito et al., 2016; Forero-Quintero et al., 2017). Furthermore, branched-chain ketoacids, which are secreted by glioblastoma cells in large amounts via MCT1, reduce phagocytic activity of tumor-associated macrophages, which in turn may promote tumor growth (Silva et al., 2017).

Lactate transport across the cell membrane is facilitated by different intracellular and extracellular carbonic anhydrases (CAs), which form transport metabolons with MCTs (Figure 1). A metabolon has been defined as a supramolecular complex of sequential metabolic enzymes and cellular structural elements in which metabolites are passed from one active site to another without complete equilibration with the bulk cellular fluids (Srere, 1985, 1987; Deitmer and Becker, 2013). Intracellular CAII accelerates transport activity of MCT1 and MCT4 twofold (but not of MCT2), when the proteins were co-expressed in *Xenopus* oocytes (Becker et al., 2005, 2010; Klier et al., 2011). CAII-mediated facilitation of lactate transport is independent from the enzyme’s catalytic activity (Becker et al., 2005, 2010; Becker and Deitmer, 2008), but requires direct binding of CAII to the MCT C-terminal tail (Stridh et al., 2012; Noor et al., 2015; Noor S.I. et al., 2018). CAII was suggested to function as a “proton antenna” for MCTs, which shuttles H^+^ between the transporter pore and surrounding protonatable buffer molecules to drive H^+^-coupled lactate flux (Becker et al., 2011; Noor et al., 2017; Noor S.I. et al., 2018). A non-enzymatic transport metabolon of MCT1 and CAII was also demonstrated in astrocytes (Stridh et al., 2012). Knockdown, but not chemical inhibition of catalytic activity, of CAII resulted in reduced lactate transport in Bergman glial cells in mouse cerebellar slices and cultured astrocytes, as measured by pH-imaging and flux measurements, respectively (Stridh et al., 2012). Furthermore, a close colocalization between MCT1 and CAII could be demonstrated in astrocyte cultures by an *in situ* proximity ligation assay, suggesting that MCT1 and CAII form a transport metabolon in astrocytes (Stridh et al., 2012).

Lactate flux is also facilitated by the extracellular CA isoforms CAIV and CAIX, the former being expressed in astrocytes and neurons (Svichar et al., 2006, 2009; Klier et al., 2011, 2014; Jamali et al., 2015). Non-enzymatic facilitation of MCT activity by extracellular CAs requires physical interaction between transporter and enzyme. In contrast to CAII, CAIV and CAIX do not bind to MCTs directly, but to the Ig1 domain of the transporters’ chaperons CD147 (for MCT1 and MCT4) and GP70 (for MCT2) (Forero-Quintero et al., 2018; Ames et al., 2019). Facilitation of lactate flux by extracellular CAs was also demonstrated in astrocytes and neurons (Svichar and Chesler, 2003). However, in contrast to experiments carried out on oocytes and cancer cells (Klier et al., 2011, 2014; Jamali et al., 2015; Ames et al., 2018), CA-mediated facilitation of lactate transport in the brain appeared to require CA catalytic activity (Svichar and Chesler, 2003).

Besides several catalytically active CA isoforms, brain cells also express three catalytically inactive carbonic anhydrase-related proteins (CARPs) VIII, X, and XI (Taniuchi et al., 2002; Aspatwar et al., 2010), which were speculated to function through interaction with other proteins (Aspatwar et al., 2014). A recent pilot study on *Xenopus* oocytes demonstrated that all three isoforms increased MCT1 transport activity, giving rise to the assumption that CARPs can play a role in the facilitation of H^+^-coupled lactate transport (Aspatwar et al., 2019), which awaits confirmation in brain cells.

## Modulation of Astrocytic Energy Metabolism by Neuronal Signals

Glycolysis in astrocytes is highly sensitive to excitatory neuronal activity. In particular glutamate and K^+^ can activate lactate production through different mechanisms and at different temporal scales. The stimulation by glutamate is mediated by the Na^+^/glutamate cotransporter and the Na^+^/K^+^-ATPase (Pellerin and Magistretti, 1994). Glutamate also stimulates GLUT1 trough a mechanism involving the Na^+^-glutamate cotransporter and the Na^+^/K^+^-ATPase (Loaiza et al., 2003; Porras et al., 2008; Bittner et al., 2011). K^+^, which is released during excitatory synaptic activity, has been associated to fast glycolytic activation in astrocytes. The astrocytic plasma membrane is highly permeable to K^+^. Astrocytes are responsible for extracellular K^+^ clearance, mediated by the contribution of pumps, transporters and ion channels such as Na^+^/K^+^-ATPase, NKCC1, and Kir4.1 (MacAulay and Zeuthen, 2012; Larsen et al., 2014; Larsen and MacAulay, 2017). K^+^-dependent astrocytic alkalinization by sodium-bicarbonate cotransport (NBCe1)-mediated HCO_3_^–^ uptake (termed Depolarization-Induced Alkalinization) has recently been identified as the signal for glycolytic activation in astrocytes *in vitro* and *in situ*, presumably due to a pH-dependent activation of phosphofructokinase 1 (PFK1) (Bittner et al., 2011; Ruminot et al., 2011, 2019; Sotelo-Hitschfeld et al., 2015; Fernández-Moncada et al., 2018; Köhler et al., 2018). PFK1 also exerts control of pyruvate production via allosteric modulation of pyruvate kinase (PK) by fructose 1,6-biphosphate (Jurica et al., 1998). Increases of extracellular K^+^ appears to be involved also in glycogen degradation in astrocytes, a phenomenon which involves a delayed rise of cAMP through the HCO_3_^–^-sensitive soluble adenylyl cyclase (sAC). sAC is activated by HCO_3_^–^, which is imported by NBCe1. Activity of NBCe1 in turn is stimulated by K^+^-induced depolarization of the cell membrane (Deitmer and Szatkowski, 1990; Choi et al., 2012; MacVicar and Choi, 2017). Recently, astrocytic NBCe1-dependent glycolytic activation has also been linked to the inhibition of mitochondrial respiration (Fernández-Moncada et al., 2018), suggesting that fast brain aerobic glycolysis may be interpreted as a strategy whereby neurons manipulate neighboring astrocytes to obtain more oxygen at the expense of oxygen supply to astrocytes.

Astrocytic membrane depolarization, induced by K^+^, also exerts fast lactate release, a phenomenon that has been attributed to the opening of a novel 37 pS lactate-permeable Cl^–^ channel, gated by cell depolarization, whose molecular identity is under current investigation (Sotelo-Hitschfeld et al., 2015). This indicates that after K^+^ stimulation, astrocytes respond with acute glycolytic activation and fast release of lactate.

## pH Regulation in Astrocytes

Since protons are pivotal for controlling different processes of energy metabolism as well as for the transport of lactate and the formation of bicarbonate, pH regulation in brain cells and brain tissue plays a prominent role. Besides supplying neurons with energy, astrocytes also contribute to pH homeostasis in the brain. Like most other cell types, astrocytes employ multiple membrane transporters, enzymes, and H^+^ buffers to maintain intracellular pH within a physiological window (pH_i_ 7.2–7.3, [H^+^]_i_ 63–50 nM). HCO_3_^–^-dependent cytosolic H^+^ buffering, fast transport of HCO_3_^–^ via NBCe1, and reversible conversion of H^+^ and HCO_3_^–^ to CO_2_ by CAII (Figure 1), render a high cytosolic H^+^ buffer strength in astrocytes (Theparambil et al., 2014, 2015; Theparambil and Deitmer, 2015). NBCe1 operates with a stoichiometry of 1Na^+^:2HCO_3_^–^ and possesses an equilibrium potential close to −74 mV in glial cells (Deitmer and Schlue, 1989). Considering the steep negative resting membrane potential of −70 to −90 mV in astrocytes, NBCe1 may operate close to its reversal potential in steady state. Therefore, changes in V_m_, pH, [HCO_3_^–^], or [Na^+^] can change NBCe1 transport direction, rendering NBCe1 an acid extruder as well as an acid loader in astrocytes (Deitmer and Szatkowski, 1990; Munsch and Deitmer, 1994; Theparambil et al., 2015, 2017). The activation or inhibition of astroglial pH-regulating mechanisms can even cause a complimentary pH change of the extracellular milieu (Deitmer, 1992; Chesler, 2003), which can have significant impact on neuronal activity, in particular attributable to the H^+^ dependence of a variety of ion channels and biochemical processes involved in synaptic transmission. Astroglial membrane depolarization by extracellular K^+^, released by neurons during activity, may stimulate HCO_3_^–^ uptake in neighboring astrocytes via inwardly directed NBCe1 (see also above). This would alkalinize astroglial cytoplasm and acidify the extracellular milieu of the brain (Deitmer and Szatkowski, 1990; Rose and Deitmer, 1994; Chesler, 2003). Inwardly directed NBCe1 may also raise cytosolic Na^+^ levels, which in turn would modulate Na^+^-dependent cellular processes in astrocytes (Theparambil et al., 2015; Noor Z.N. et al., 2018). Interestingly, pH-dependent astroglial Ca^2+^ signaling via stimulation of reversed Na^+^/Ca^2+^ exchanger activity (NCX) in brainstem astrocytes is mediated by a robust cytosolic Na^+^ rise, derived from inwardly directed NBCe1 activity, which is important for the control of adaptive breathing (Gourine et al., 2010; Turovsky et al., 2016).

Since NBCe1 activity in astrocytes is bi-directional, export of HCO_3_^–^ via outwardly directed NBCe1 can be significant for ensuring high H^+^ buffer strength in the narrow extracellular spaces. Astroglial HCO_3_^–^ secretion contributes to the maintenance of pH_e_ within the physiological window, and thus is fundamental for the control of H^+^-dependent neuronal excitability (Deitmer, 1992; Chesler, 2003; Theparambil et al., 2017). The secretion of HCO_3_^–^ via NBCe1 (Deitmer, 1991) could also facilitate shuttling of H^+^-coupled substrates, like lactate and glutamine, to the brain extracellular spaces, since co-transported H^+^ is effectively buffered by HCO_3_^–^ (Becker and Deitmer, 2004; Becker et al., 2004; Wendel et al., 2008). Astrocytes are also reported to express HCO_3_^–^-independent pH regulators, like the Na^+^/H^+^ exchanger NHE1 and vacuolar type ATPase (V-ATPase) (Chesler, 2003; Hansen et al., 2015). The functional expression of NHE1 is found in astrocytes from all brain regions, but V-ATPase activity is only reported in astrocytes from optic nerve preparations and in hippocampal cell cultures (Pappas and Ransom, 1993; Hansen et al., 2015). This strongly supports the conclusion that astroglial pH regulatory mechanisms are linked to vital neurological functions. Indeed, a recent study showed that astroglial-specific deletion of NHE1 protects from ischemic stroke-induced brain damage by reducing astrogliosis, blood brain barrier damage and infarction (Begum et al., 2018).

## Conclusion and Perspectives

The results summarized here indicate that energy supply to the brain includes not only diffusion and uptake of glucose into the cells, but also a modulated transfer of lactate from astrocytes to neurons. Since astrocytes, but not neurons, have direct access to blood capillaries and hence to blood-born glucose, they may take up more glucose than neurons, although they require less energy for physiological functioning. Hence, astrocytes can up-regulate glycolysis and glycogenolysis to sacrifice the glycolytic end-product by transferring lactate to neurons, thereby reducing their oxidative metabolism to allow neurons to use more of the available oxygen to boost their own oxidative energy production. When active, neurons communicate to their neighboring astrocytes their need for enhanced energy supply by releasing glutamate and/or K^+^. The production and the release of lactate by astrocytes are thus directly linked to neuronal activity and modulated by a variety of processes, like membrane depolarization, transmitter release, H^+^ buffering, and H^+^ antennae of CAs. Energy dynamics in the brain thereby exploit cooperative processes of neurons and astrocytes to meet the high energy requirements of neuronal synapses and to ensure high-speed information processing in the brain.

## Author Contributions

All authors listed have made a substantial, direct and intellectual contribution to the work, and approved it for publication.

## Conflict of Interest

The authors declare that the research was conducted in the absence of any commercial or financial relationships that could be construed as a potential conflict of interest.
